# Association of IS*Vsa3* with Multidrug Resistance in *Salmonella enterica* Isolates from Cattle (*Bos taurus*)

**DOI:** 10.3390/microorganisms11030631

**Published:** 2023-03-01

**Authors:** Gentry L. Lewis, Robert J. Fenton, Etsuko N. Moriyama, John Dustin Loy, Rodney A. Moxley

**Affiliations:** 1 School of Veterinary Medicine & Biomedical Sciences, University of Nebraska-Lincoln, Lincoln, NE 68583, USA; 2 School of Biological Sciences and Center for Plant Science Innovation, University of Nebraska-Lincoln, Lincoln, NE 68588, USA

**Keywords:** *Salmonella enterica*, antimicrobial resistance, cattle, multidrug resistance, whole-genome sequence, mobile genetic element, IS*CR*, IS*Vsa3*

## Abstract

*Salmonella enterica* is, globally, an important cause of human illness with beef being a significant attributable source. In the human patient, systemic *Salmonella* infection requires antibiotic therapy, and when strains are multidrug resistant (MDR), no effective treatment may be available. MDR in bacteria is often associated with the presence of mobile genetic elements (MGE) that mediate horizontal spread of antimicrobial resistance (AMR) genes. In this study, we sought to determine the potential relationship of MDR in bovine *Salmonella* isolates with MGE. The present study involved 111 bovine *Salmonella* isolates obtained collectively from specimens derived from healthy cattle or their environments at Midwestern U.S. feedyards (2000–2001, *n* = 19), or specimens from sick cattle submitted to the Nebraska Veterinary Diagnostic Center (2010–2020, *n* = 92). Phenotypically, 33/111 isolates (29.7%) were MDR (resistant to ≥3 drug classes). Based on whole-genome sequencing (WGS; *n* = 41) and PCR (*n* = 111), a MDR phenotype was strongly associated (OR = 186; *p* < 0.0001) with carriage of IS*Vsa3*, an IS*91*-like Family transposase. In all 41 isolates analyzed by WGS ((31 MDR and 10 non-MDR (resistant to 0–2 antibiotic classes)), MDR genes were associated with carriage of IS*Vsa3*, most often on an IncC type plasmid carrying *bla*_CMY-2_. The typical arrangement was *floR*, *tet(A)*, *aph(6)-Id*, *aph(3″)-Ib*, and *sul2* flanked by IS*Vsa3*. These results suggest that AMR genes in MDR *S. enterica* isolates of cattle are frequently associated with IS*Vsa3* and carried on IncC plasmids. Further research is needed to better understand the role of IS*Vsa3* in dissemination of MDR *Salmonella* strains.

## 1. Introduction

*Salmonella enterica* subsp. *enterica* (*S. enterica*) is, globally, an important cause of human illness, and in the United States (U.S.), it is estimated to cause 1.35 million infections, 26,500 hospitalizations, and 420 deaths each year [1]. Although ranking behind seeded vegetables, eggs, and poultry, beef is a significant attributable source of *S. enterica* [2]. Specific to nontyphoidal *Salmonella*, beef ranks 14th, 8th, and 8th out of the top 37 pathogen–food pairs in burden of illness in the U.S. in terms of number of illnesses, basic cost, and economic cost, respectively [3]. In the human patient, systemic *Salmonella* infection requires antibiotic therapy [4,5], and when the strain is MDR (resistant to ≥3 antibiotic classes) [6], the case is particularly problematic. First-line antibiotic therapy for systemic *Salmonella* infections in humans includes third-generation cephalosporins (e.g., ceftriaxone), fluoroquinolones (e.g., ciprofloxacin), and macrolides (e.g., azithromycin) [4,5]. However, fluoroquinolones have adverse side effects in children and pregnant women [7,8], in which case ceftriaxone and azithromycin are the drugs of choice. Unfortunately, ceftriaxone and ciprofloxacin resistance in human *Salmonella* isolates has increased in recent years [9,10].

*S. enterica* is also a primary pathogen in cattle, mainly causing enteritis in calves between 2 and 6 weeks of age, but can also cause enteritis, pneumonia, and abortions in adult animals, with most clinical infections associated with *S*. Dublin and *S*. Typhimurium [11]. However, more than 143 serotypes have been found in cattle lacking clinical signs of illness, indicating a large reservoir of diversity in populations [12]. *S. enterica* is often associated with subclinical infections, but may have a morbidity of over 50% in calves, with a case–fatality rate approaching 100% without treatment [13]. The most common resistance pattern in *S.* Typhimurium is ampicillin, chloramphenicol, streptomycin, sulfamethoxazole, and tetracycline (ACSSuT) [13]. This MDR pattern has, historically, most often corresponded to a clone of *S.* Typhimurium known as bacteriophage definitive type (DT) 104 (DT104), which often causes severe disease in both animals and humans [14].

The prevalence of AMR and MDR in *Salmonella* isolated from cattle, their environments, and beef products has increased over the past few decades. The prevalence of MDR *S.* Newport isolates from cattle, their environments, and beef products in Canada increased from 2000 to 2002 compared to before 2000, with 50% of isolates resistant to at least 11 antimicrobials, including the extended-spectrum cephalosporins [15]. In a study of clinical *Salmonella* isolates from cattle in Alberta, Canada from 2006 to 2014, *S.* Typhimurium and *S.* Dublin constituted the majority of isolates, and the prevalence of MDR was 89.1% and 93.8%, respectively [13]. In *S.* Dublin isolates from cattle in California, compared to isolates from 1993 to 1999, there was an increase in resistance among quinolone and cephalosporin drugs from 2006 to 2010, and an increase in the number of isolates with an MDR profile [16].

In bacteria, the spread of AMR genes is mainly the result of mobile genetic elements (MGE), which enable intracellular and intercellular movement of DNA, e.g., insertion sequences (IS), transposons (Tn), integrons (In), plasmids, integrative conjugative elements (ICE), and integrative mobilizable elements (IME) [17,18]. DNA containing AMR genes is also spread intercellularly via transduction and transformation mechanisms [17]. Many MDR *Salmonella* such as *S.* Typhimurium DT104 contain an IME known as a *Salmonella* Genomic Island (SGI) [19]. SGI1 variants contain different combinations of genes responsible for the ACSSuT phenotype, and, in addition, those for resistance to florfenicol, gentamycin, spectinomycin, tobramycin, and trimethoprim [19]. To date, 12 variants of SGI1 are recognized among at least 16 different *Salmonella* serotypes [19].

Recent studies have shown that ICE*Mh1* and -like elements in respiratory pathogens of the *Pasteurellaceae* family readily spread among each other in cases of bovine respiratory disease (BRD), and have the potential to spread into *Salmonella* [20]. Spread of these ICEs is especially evident in outbreaks of BRD in high-risk stocker and feedlot calves following metaphylactic and therapeutic administration of antibiotics [21,22,23,24]. ICE*Mh1* in *Mannheimia haemolytica* and ICE*Mh1*-like elements such as ICE*Pmu1* in *Pasteurella multocida* are fully transmissible and proven to integrate into *P. multocida*, *M. haemolytica*, and *Escherichia coli* recipients. They potentially integrate into *Salmonella* based on DNA analytical evidence of the target integration site in the chromosome. Each of these ICEs transmits a potent arsenal of antibiotic resistance. ICE*Pmu1* contains 12 resistance genes: *strA* and *strB* (conferring resistance to streptomycin), *aphA1* (gentamicin), *sul2* (sulfonamides), *tet(H)* (tetracyclines), *floR* (phenicols), *erm(42)* (macrolides and lincosamides), *aadB* (gentamicin), *aadA15* (streptomycin and spectinomycin), *bla*_OXA-2_ (β-lactams), and *msr(E)* and *mph(E)* (macrolides). ICE*Mh1* contains five resistance genes: *strA* (*aph(3″)-Ib*), *strB* (*aph(6)-Id*), *aphA1*, *sul2*, and *tet(H)*. Antibiotics for metaphylaxis (control) of BRD could potentially select for AMR *Salmonella* secondary to selection for respiratory pathogens carrying ICE*Mh1* and ICE*Mh1*-like elements. In addition, they could directly select for AMR *Salmonella* strains that contain SGI1 variants.

We hypothesize that ICE*Mh1*, ICE*Pmu1*, or other ICE*Mh1*-like elements occur in *Salmonella* isolates. To our knowledge, no studies have investigated this hypothesis. The objectives of this study were to determine, in a set of *Salmonella enterica* isolates from cattle or their environments, the frequency of: (1) AMR genes typically associated with ICE*Mh1* and ICE*Mh1*-like integrative conjugative elements; (2) *Salmonella* Genomic Island 1 (SGI1) variants and their associated AMR genes; and (3) other mobile genetic elements and their potential association with MDR.

## 2. Materials and Methods

### 2.1. Bacterial Strains

#### 2.1.1. *S. enterica* Isolates from Midwestern U.S. Feedyards from 2000 to 2001

All *S. enterica* strains used in this study (*n* = 111) were isolates from 2000 to 2001 (*n* = 19) or 2011 to 2020 (*n* = 92). The 19 isolates from 2000 to 2001 were a subset of 530 isolates from feedlot beef cattle feces or their pen environments in Midwestern U.S. feedyards [25] (Table 1). All 530 isolates had been serotyped and tested for antimicrobial susceptibility phenotype in 2006 using a standardized National Animal Resistance Monitoring System (NARMS) protocol, and also were tested by PCR for class 1 integron genes [26]. Of the 530 isolates from that study, 0 were positive for class 1 integron genes by PCR; however, based on the NARMS 2006 results, 13 were MDR (Table 1). These 13 MDR isolates were selected for inclusion in the present study; 6 other isolates that were resistant to only 1 antimicrobial that originated from the same sample or pen of cattle were also included for whole-genome sequence (WGS) and/or other test comparisons. Immediately prior to this study, all 19 strains were retested for antimicrobial susceptibility using the Sensititre BOPO7F veterinary plates (Thermo Fisher Scientific, Waltham, MA, USA) to provide additional data regarding their susceptibility to antimicrobials in current use for cattle, including those relevant for respiratory pathogens (Table 1). Based on the BOPO7F results, 2 of the 13 strains had become pan-susceptible in storage. Eleven of the 13 strains, including the 2 that had become pan-susceptible, and 6 non-MDR strains from the same study [26], were selected for WGS (Table 1).

#### 2.1.2. Bacterial Strains Used as Controls for Multiplex qPCR (mqPCR) Assay for Detection of AMR Genes Associated with ICE*Pmu1* and ICE*Mh1*

*M. haemolytica* strain 2308 was isolated from a bovine clinical sample by the NVDC from a diagnostic submission. This isolate had previously been determined by Sanger sequencing to contain eight AMR genes associated with ICE*Pmu1*, namely *aphA1*, *sul2*, *tetR(H)*, *floR*, *erm(42)*, *bla*_OXA2_, *msr(E)*, and *mph(E)*, with four of these genes also associated with ICE*Mh1*; all eight genes had been detected by a mqPCR assay (described below) that had been co-developed by one of the authors (J.D.L.) [27], hence *M. haemolytica* strain 2308 was validated for use as positive control for this mqPCR assay. *E. coli* strain 25922 (American Type Culture Collection) is a laboratory strain that does not possess these genes, and was used as a negative control for the mqPCR assay (described below).

#### 2.1.3. Isolates from Nebraska Veterinary Diagnostic Center from 2011 to 2020

A second source of *S. enterica* strains was accessions from cattle systems to the Nebraska Veterinary Diagnostic Center (NVDC) during the period of 2011–2020. Of 98 *Salmonella* isolates identified from these accessions, 92 were viable from frozen stocks and included in the present study. Of these 92 isolates, 83 were from 42 of the 93 counties in Nebraska; 5 were from Missouri; and 1 each was from California, Colorado, Idaho, and Iowa. The 92 isolates had been serotyped previously as part of the diagnostic process, and included 27 different serotypes with *S.* Typhimurium (including 3 var. 5-) (*n* = 18), *S.* Newport (*n* = 13), *S.* Dublin (*n* = 10), *S.* Montevideo (*n* = 8), and *S.* Muenster (*n* = 7) constituting the 5 most prevalent and 60.9% (56/92) of the total (Table S1). A signalment (e.g., age, sex) and clinical history (e.g., diarrhea, abortion) and/or pathology data (e.g., enteritis, pneumonia) was provided in association with 84 (91.3%) and 78 (84.8%) of the cases, respectively. Salmonellae were most commonly isolated from accessions involving unweaned/neonatal calves (38.0%) and cows/heifers (29.3%) (Table S2). Overall, based on the clinical history and accompanying laboratory results, the *Salmonella* isolates were associated with disease (i.e., salmonellosis) in 81.5% of the accessions. Diarrheal disease (enteritis/colitis) and pneumonia were the most common manifestations, reported in 53.3% and 20.7% of the accessions, respectively.

The NVDC isolates were subjected to antimicrobial susceptibility testing either at the time of the accession or immediately prior to this study, if they had not been previously tested. Testing was conducted at the time of the accession either using the Sensititre BOPO6F or BOPO7F plate formats (Thermo Fisher Scientific Waltham, MA, USA), or immediately prior to this study using the Sensititre BOPO7F. Antimicrobial susceptibility testing was conducted using Clinical and Laboratory Standards Institute (CLSI, Annapolis, MD, USA) methods and recommended quality control strains for the broth microdilution assay [28]. Veterinary specific breakpoints were applied when available [29]. Of the 92 NVDC isolates, 22 (23.9%) were MDR, with serotypes *S.* Dublin (*n* = 10) and *S.* Newport (*n* = 5) combined representing 68.1% (15/22) of the MDR isolates (Table 2). Of the 22 MDR isolates, resistance was most frequent to florfenicol and sulfadimethoxine (95.4% each), followed sequentially by 1 or more of the tetracyclines (chlortetracycline, oxytetracycline, and/or tetracycline; 90.9%), ceftiofur (77.3%), and a fluoroquinolone (danofloxacin and/or enrofloxacin; 40.9%). Resistance to macrolides (clindamycin, gamithromycin, tiamulin, tilmicosin, tildipirosin tulathromycin, and tylosin tartrate) was considered intrinsic and not reported. Aminoglycoside test results (gentamicin, neomycin, and spectinomycin) were also largely excluded since breakpoints and assessments of susceptibility or resistance for *Salmonella* are difficult to determine. Twenty of the 22 MDR strains were selected for WGS.

### 2.2. Culture of Bacterial Strains and DNA Preparation

Frozen stock cultures (−80 °C) of *Salmonella* strains were streaked for isolation onto Luria Broth (Miller*,* Appleton, WI, USA; LB) Agar (Becton, Dickinson and Company, Sparks, MD, USA) and incubated 18–24 h at 37 °C. A single well-isolated colony was used to inoculate 5 mL LB, and this culture was incubated 24 h, stationary at 37 °C. A 2-mL aliquot of broth culture was moved into the GeneJET DNA Genomic Purification Kit (Thermo Fisher Scientific, Waltham, MA, USA) to prepare the DNA template for mqPCR reactions. Extractions were performed according to the manufacturer’s protocol. Frozen stock cultures (−80 °C) of *Mannhemia haemolytica* control strains were streaked for isolation onto Trypticase Soy Agar (TSA) with 5% Sheep Blood (BD) and incubated 18–24 h at 37 °C. A single well-isolated colony was used to inoculate 50 mL Brain Heart Infusion (BHI) in a 250 mL Erlenmeyer flask, aerated at 150 rpm for 24 h, at 37 °C. A 2 mL aliquot of broth culture was transferred into the GeneJET DNA Genomic Purification Kit (Thermo Fisher Scientific, Waltham, MA, USA) to prepare the DNA template for mqPCR reactions. Extractions were performed according to the manufacturer’s protocol. Purified DNA concentration for each isolate was determined via NanoDrop One (Thermo Fisher Scientific, Waltham, MA, USA) with *M. haemolytica* strain 2308 and *E. coli* strain 29522 as the positive and negative organismal controls, respectively, for the mqPCR assay (described below) and Invitrogen UltraPure Water (Thermo Fisher Scientific, Waltham, MA, USA) in place of DNA as the negative reaction control.

### 2.3. mqPCR

The mqPCR assay and targets were in part based on previous work with applications to BRD pathogens [27]. Four-plex mqPCR (25-µL reaction) assays included targets, reagents, and primers as described in Table S4. The mqPCR reaction consisted of 12.5 µL of 2X Quantifast Multiplex PCR Master Mix (Qiagen), 1.0 μL of each primer probe mix (4 μL total) containing F (10 μM), R (10 μM), P (5 μM), 9.5 μL Invitrogen UltraPure Water (Thermo Fisher Scientific, Waltham, MA, USA), and 2.0 µL (5 ng/μL) of template DNA. mqPCR reactions were carried out in a CFX96 (Bio-Rad, Hercules, CA, USA) under the following conditions: 95 °C for 5 min, then 45 cycles of 95 °C for 15 s, 59 °C for 40 s.

### 2.4. Endpoint PCR

Frozen stock cultures (−80 °C) of bacterial strains were streaked for isolation onto Luria Broth (Miller; LB) Agar (Becton, Dickinson and Company, Sparks, MD) and incubated 18–24 h at 37 °C. A single well-isolated colony was picked, suspended in 50 μL of UltraPure Water, and heated at 95 °C in the thermocycler for 10 min. A 2.0 µL aliquot of this DNA template was used in the 25-µL PCR reaction. Individual 25-µL reaction PCR assays were conducted using primer pairs as shown in Table S5 [27,30,31]. The PCR reaction consisted of 2.5 µL 10X ThermoPol Reaction Buffer, 1.0 μL of each forward and reverse primer (10 μM each, 2 μL total), 0.5 µL dNTP mix (10 mM each dNTP), 0.25 µL NEB Taq DNA Polymerase (New England Biolabs, Ipswich, MA, USA), 17.75 µL UltraPure Water, and 2.0 µL (5 ng/μL) of template DNA. UltraPure Water volume was adjusted for multiplex PCR reactions. PCR reactions were carried out in a T100 Thermal Cycler (Bio-Rad, Hercules, CA, USA) under the following conditions: 95 °C for 5 min, then 30 cycles of 95 °C for 15 s, 59 °C for 40, 72 °C for 30 s, and a final elongation at 72 °C for 7 min. The positive organismal control was *S.* Typhimurium strain RM014 (this study), and the negative reaction control was UltraPure Water in place of DNA. PCR reactions were run on 1.2% agarose TAE gels stained with ethidium bromide and visualized on a ChemiDoc MP Imager (Bio-Rad, Hercules, CA, USA).

### 2.5. WGS

WGS was conducted at the Iowa State University Veterinary Diagnostic Laboratory by Dr. Ganwu Li. Pure cultures were used for DNA extraction with the MagMAX Pathogen RNA/DNA Kit (Thermo Fisher Scientific, Waltham, MA, USA) and a Kingfisher Flex instrument (Thermo Fisher Scientific, Waltham, MA, USA) following the manufacturer’s instructions. Nucleic acid was eluted into 45 μL of elution buffer and stored at −80 °C until used. Indexed genomic libraries were prepared using the Nextera XT DNA Library Prep Kit (Illumina, San Diego, CA, USA) and quantified by the Qubit fluorometer dsDNA HS kit (Thermo Fisher Scientific, Waltham, MA, USA). The library was sequenced on an Illumina MiSeq platform (Illumina, San Diego, CA, USA) with either MiSeq Reagent Kit v2 (500 cycle) or MiSeq^®^ Reagent Kit v3 (600 cycle). For Nanopore sequencing, pure cultures were submitted to Iowa State University DNA Facility (Ames, IA, USA) for DNA extraction and Nanopore sequencing on the Oxford Nanopore GridION X5 (Oxford Nanopore Technologies, Oxfordshire, England).

### 2.6. Sequencing Quality Control and Genome Assembly

Following sequencing, Illumina short read quality was assessed using FastQC v0.11.7 (Babraham Bioinformatics, 2018, Babraham Institute, Cambridge, UK). BBDuk (v37.62) was used to trim adapters from the lllumina short reads, and any short reads with average quality score (Q score) below 30 were discarded. The porechop (v0.2.4) was used to trim adapters from the Nanopore long reads. The average sequencing depth of each isolate was estimated by dividing the total length of its cleaned reads by the genome size. Additionally, Illumina reads and Nanopore long reads from each isolate were hybrid de novo assembled using Unicycler (v0.4.9). Isolates with unclosed genomes were reassembled by Raven (v1.5.1) with Nanopore long reads and then polished by Pilon (v1.24) with Illumina short reads. All isolates sequenced in this study had >95× depth and N50 > 4.6 Mb with genome sizes between 4.6 and 5.0 Mb (Megabases; million bases). All the genomes were closed.

### 2.7. GenBank Accessions

All WGS data on the 41 isolates is available under NCBI BioProject PRJNA929056.

### 2.8. Bioinformatic and Statistical Analyses

Genomic DNA sequence similarity searches were conducted using BLAST+2.11.0 [32]. Genomes were annotated using NCBI Prokaryotic Genome Annotation Pipeline (PGAP) 2021-07-01.build5508 (https://www.ncbi.nlm.nih.gov/genome/annotation_prok/ accessed on 1 July 2021) [33]. Sequence visualization and analysis was conducted using Geneious Prime 2021.2.2 (https://www.geneious.com, accessed on 1 July 2021). AMR gene identification and verification was conducted using CARD 3.1.3 (https://card.mcmaster.ca/, accessed on 1 July 2021) [34] and ResFinder 4.1 2021-08-16 [35]. Maximum-likelihood phylogenies were estimated using PhyML 3.2 [36]. Sequence alignments were conducted using Muscle 3.8.425 [37]. Pan-genome analyses were conducted using Roary 3.13.0 [38]. Core phylogeny with metadata analysis was conducted using Phandango 1.3.0 [39]. The association between an MDR phenotype and IS*Vsa3* genotype (combined results of PCR and WGS) in the 111 *S. enterica* isolates was determined by calculation of the odds ratio (OR) with 95% confidence interval (CI) and *p* value (<0.05 interpreted as significant) using MedCalc^®^ statistical software, Version 20.218 [40].

## 3. Results

### 3.1. Frequency of Antimicrobial Resistance (AMR) Genes Typically Associated with ICEMh1 and ICEMh1-like Integrative Conjugative Clements

Total genomic DNA extracted from all *S. enterica* isolates listed in Table 1 and Table S3 except RM001, RM002, RM003, RM004, RM005, and RM006 (*n* = 105) was tested by mqPCR for AMR genes located on ICE*Mh1* and ICE*Mh1*-like elements, including *tetR(H)* (tetracycline), *msr* (macrolide), *mph* (macrolide), *erm* (macrolide), *floR* (phenicol), *sul2* (sulfonamide), *bla*_OXA2_ (β-lactamase), and *aphA1* (aminoglycoside). Additional genes tested for by endpoint PCR in these 105 isolates included *bla*_CMY-2_ (β-lactamase), IS*Vsa3* (IS91-like Family transposase), *tet(A)* (tetracycline), and *sul2* (sulfonamide). By mqPCR, the frequency of isolates positive was *floR*, 30.5%; *sul2*, 30.5%; and *aphA*1, 4.8%, whereas 0% were positive for *tetR(H)*, *erm*, *msrE*, *mphE*, or *bla*_oxa2_ (Table S6). Hence, the isolates were negative for most AMR genes carried by ICE*Mh1* and ICE*Mh1*-like elements, suggesting that *floR* and *sul2* were possibly associated with one or more other mobile genetic elements. Endpoint PCR assays revealed that 25.7%, 30.5%, and 28.6% were positive for *bla*_CMY-2_, IS*Vsa3*, and *tet(A)*, respectively (Table S6). Based on mqPCR and endpoint PCR results, the correlation coefficients (CORREL, Excel 2016) were 1.00 for *floR* versus IS*Vsa3* and 0.96 for *sul2* versus IS*Vsa3*, suggesting that *floR* and *sul2* were associated with IS*Vsa3* instead of ICE*Mh1* and ICE*Mh1*-like elements.

### 3.2. Frequency of Salmonella Genomic Island 1 (SGI1) and SGI1 Variants

In our previous study [26], 0 of 530 beef feedlot pen *S. enterica* isolates was positive by endpoint PCR for the class 1 integron gene (*intI1*), suggesting that SGI1 was not involved in MDR. We again analyzed the 19 MDR isolates from that study (Table 1) and the 22 NVDC MDR isolates (Table 2) for *intI1* by WGS. By WGS, consistent with the previous study [25], 0 of 9 MDR feedlot pen isolates tested were positive for *intI1*; however, 3 of 22 WGS NVDC isolates (13.6%) and 3 of 41 WGS isolates (7.3%) overall were positive for *intI1*; all 3 *intI1* positive isolates were MDR (Table S6). These three isolates (RM055, RM074, and RM101), in addition to *intI1*, had *qacEΔ1* and *sul1*, and one (RM055) also had *aadA2*. Interestingly, none of these genes, which are markers of SGI1, were on the chromosome; all were on a plasmid that also carried IS*Vsa3* (Table S6).

### 3.3. Other Mobile Genetic Elements, Their Genomic Locations, and MDR Association

**Figure 1:**
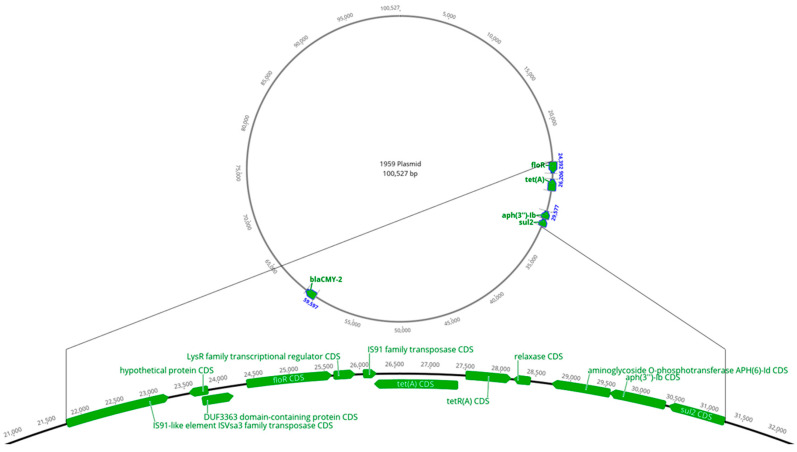
Location of IS*Vsa3* and flanking antimicrobial resistance genes on a *bla*_CMY-2_ positive IncC plasmid of *Salmonella* Uganda isolate 1959.

**Figure 2:**
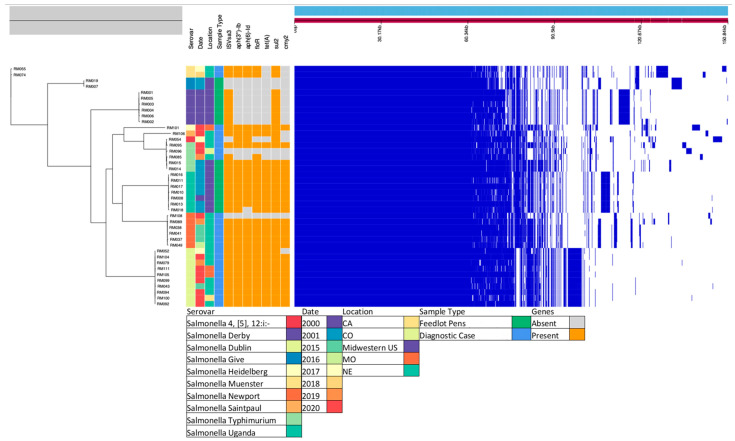
Maximum-likelihood phylogenetic tree constructed from an alignment of core genes and selected metadata. The right panel shows a broad overview of the presence/absence of all genes sequenced for each strain. The phylogeny depicts the evolutionary relationship of the isolates sequenced. Ten serovars were represented in the metadata of the 41 *S. enterica* isolates subjected to WGS. These diagnostic cases and feedlot pen strains were isolated in eight different years spanning a ten-year period from different areas in the US. The presence and absence of IS*VSa3* and pertinent genes can be visualized in orange and grey, respectively (presence of IS*Vsa3*, 36/41; *aph(3″)-Ib*, 30/41; *aph(6)-Id*, 29/41; *floR*, 30/41; *tetA*, 27/41; *sul2*, 36/41; *cmy2*, 25/41). The core gene alignment to the right (in dark blue) reflects the conservation and diversion in these genomes.

By WGS, all 41 isolates (100%) had the following AMR genes on the chromosome: *aac(6′)*, *aac(6′)-Iaa*, and *aadA* (all involved in aminoglycoside resistance); *ampH* (a penicillin-binding protein; PBP); *bacA* (involved in bacitracin resistance); and *mrdA* (a PBP known to confer reduced susceptibility to carbapenems) (Table S6). However, the presence of these genes was not associated with AMR for the respective antibiotic classes. Instead, resistance was associated with the following: aminoglycoside with *aph(3″)-Ib* and *aph(6)-Id*; phenicol with *floR*; tetracycline with *tet(A)*; sulfonamide with *sul2*; and β-lactam with *bla*_CMY-2_ (Table S6). A total of 25 out of 41 isolates were positive for all 5 genes: *floR*, *tet(A)*, *aph(6)-Id*, *aph(3″)-Ib*, and *sul2* (Table 3), typically arranged in that order and flanked by IS*Vsa3* (Figure 1 and Figure 2). Hence, an MDR phenotype was predominantly associated with carriage of IS*Vsa3* in which the genes were most often located on an IncC type plasmid that also carried *bla*_CMY-2_ (Table 4; Figure 1). Based on the combined results of endpoint PCR and WGS, 31/33 (93.9%) MDR isolates were positive for IS*Vsa3*, whereas 6/78 non-MDR isolates (7.7%) were positive for IS*Vsa3* (OR = 186.00, *p* < 0.0001; Table 5). In addition, based on combined data from endpoint PCR and WGS, of the 111 isolates, the number (percentage) positive for the above-mentioned genes was: 38 (34.2%) for IS*Vsa3*; 27 (24.3%) for *bla*_CMY-2_; 33 (29.7%) for *floR*; 30 (27.0%) for *tet(A)*; 38 (34.2%) for *sul2*; and 31 (27.9%) for both *aph(3″)-Ib* and *aph(6)-Id* (Tables S6 and S7). The serotypes in which IS*Vsa3* was found included *S.* Derby, *S.* Uganda, *S.* Typhimurium, *S.* Newport, *S.* Dublin, *S.* Muenster, *S.* Heidelberg, and *S.* Saintpaul (Table S6).

### 3.4. Core Gene Alignment and Metadata

A phylogenetic tree was constructed from an alignment of core genes and selected metadata, depicting the evolutionary relationship of the isolates sequenced (Figure 2). The core gene alignment reflected the conservation and diversion in these genomes.

### 3.5. ISVsa3 and Associated AMR Genetic Segment Alignment

An alignment of the genetic segment of the 36 sequenced isolates carrying IS*Vsa3* and associated AMR genes is shown in Figure S1.

## 4. Discussion

In this study, the first objective was to address the hypothesis that ICE*Mh1*, ICE*Pmu1* or other ICE*Mh1*-like elements occur in *Salmonella* isolates. We found no evidence of these BRD pathogen-associated ICE elements in our collection of 111 isolates. It is possible that one or more of these ICE elements might have been detected if more isolates were tested, and if we had tested isolates representing a more widespread area of the country, different production settings, and cattle that had been subjected to metaphylactic treatment for BRD. A number of studies have assessed the effects of metaphylactic regimens for BRD on the prevalence and selection for AMR *Salmonella* in field trials. A randomized controlled longitudinal study that followed cattle through the entire feeding period to harvest found that one dose of tulathromycin administered to healthy cattle at feedlot arrival did not result in an increase in the prevalence or AMR of *Salmonella* [41]. We hypothesize that detection of ICE*Mh1*, ICE*Pmu1*, or other ICE*Mh1*-like elements in *Salmonella* would be more likely if one were to culture large numbers of fecal or other samples from BRD high-risk calves having respiratory colonization with ICE*Mh1*-positive *M. haemolytica* [21,22,23,24], and especially following metaphylactic treatment, but this was beyond the scope of our study. Information concerning antimicrobial treatment was not available, and that concerning the signalment and clinical history was also limited.

Our second objective was to address whether SGI1 or its variants were associated with MDR, and we found that only 3 MDR isolates (7.3%) had SGI1 genes, and in these isolates, the genes were carried on a plasmid that also carried IS*Vsa3*. One of these three isolates (RM101) carried SGI1 genes on an IncC plasmid. SGI1 cannot transfer itself into a new host because it does not carry a full set of conjugation genes, but it is mobilizable, and can be transferred if an IncC plasmid is present in the donor [19]. SGI1 only excises from the chromosome in the presence of a helper plasmid [42], and although SGI1 is known to modify and use the conjugation apparatus encoded by IncC, the two (SGI1 and IncC) are incompatible [42]. SGI1 destabilizes IncA and IncC plasmids after a few generations and, conversely, the presence of an IncC plasmid enhances the recombination rate within SGI1, leading to the generation of SGI1 deletion variants [42]. Interestingly, in our study, 20 of 29 WGS isolates (69.0%) that, collectively, had 4 or 5 AMR genes carried them on an IncC plasmid.

Our third objective was to determine the frequency of other MGE and their association with MDR, which yielded the main finding of the study: MDR was strongly associated with the presence of IS*Vsa3* (IS91-like Family transposase). This appears to be a novel finding, although other investigators recently reported similar genetic and phenotypic AMR profiles in 15 MDR *S.* Dublin isolates from retail meat and human patients [43]. In that study, nothing was stated about IS*Vsa3*, but when we searched the associated NCBI BioProject PRJNA357723 sequence data, we found IS*Vsa3* (also listed as IS*91*-like element) in 12 of the 15 isolates. Besides our finding that IS*Vsa3* was strongly associated with MDR in *Salmonella*, our results extend these findings in that IS*Vsa3* was found in MDR isolates in seven other serotypes besides *S.* Dublin.

Previous studies have shown that IS*Vsa3* was first identified in the fish pathogen *Vibrio salmonicida* [44], and made its way into other fish pathogens, e.g., *Edwardsiella piscicida* [45], and further into pathogens isolated from other animals and humans, e.g., *Salmonella* Choleraesuis and *Acinetobacter baumannii* [44], carrying with it high-level resistance to antibiotics such as the tetracyclines. IS*Vsa3* is frequently found on conjugative plasmids [44] and poses a significant threat to spread AMR.

IS*Vsa3* is an IS*91*-like MGE, referred to as an Insertion Sequence Common Region (IS*CR*) [46,47]. In our study, IS*Vsa3* was found to be in a conserved relationship with *floR*, *tet(A)*, *aph(6)-Id*, *aph(3″)-Ib*, and *sul2* in 72.2% (26 of 36 IS*Vsa3*-positive) of the WGS *S. enterica* isolates. Due to this insertion sequence families’ unique method of transposition, they are capable of and frequently responsible for mobilizing many classes of AMR genes, and are considered an evolutionary feature of IncC plasmids [47,48]. In most positive strains in our study, this IS*CR* was located on plasmids, and particularly IncC, while only 3 of those carrying IS*Vsa3* and the associated AMR genes were found on the chromosome. The strong association of IS*Vsa3* with MDR in the *Salmonella* isolates in our study and the knowledge that IS*CR* frequently assemble multiple AMR genes and transpose them into conjugatable plasmids suggests this particular transposon poses a significant threat to increasing MDR. Further research is needed to better understand the role of IS*Vsa3* in dissemination of MDR in *Salmonella*.

## Figures and Tables

**Table 1:** *Salmonella enterica* isolates from 2000 to 2001 from feedlot beef cattle feces or pen environments used in this study.

Isolate	Date	Feedyard /Pen ^1^	Sample ^2^	Serotype	NARMS (2006) ^3^	BOPO6/7F (2020) ^4^	WGS ^6^
RM001	19 September 2000	1/655c	Rope (R6)	Derby	FIS	ND ^5^	+
RM002	19 September 2000	1/655c	Water (W2)	Derby	FIS	ND	+
RM003	30 October 2000	1/669	Rope (R3)	Derby	FIS	ND	+
RM004	30 October 2000	1/669	Rope (R6)	Derby	FIS	ND	+
RM005	30 October 2000	1/655b	Rope (R1)	Derby	FIS	ND	+
RM006	30 October 2000	1/655b	Rope (R3)	Derby	FIS	ND	+
RM007	17 September 2001	2/822b	Water (W2)	Give	CHL, FIS, KAN, NAL, STR, TET	Pan-susceptible	+
RM008	17 September 2001	3/154	Feces (F1A)	Uganda	AMP, AUG2, AXO, CHL, FIS, FOX, STR, TET, XNL	AMP, FFN, SDM, TET, XNL	+
RM009	17 September 2001	3/154	Feces (F1A)	Uganda	AMP, AUG2, AXO, CHL, FIS, FOX, STR, TET, XNL	AMP, FFN, SDM, TET, XNL	-
RM010	17 September 2001	3/154	Feces (F1A)	Uganda	AMP, AUG2, AXO, CHL, FIS, FOX, STR, TET, XNL	AMP, FFN, SDM, TET, XNL	+
RM011	17 September 2001	3/157	Rope (R1)	Uganda	AMP, AUG2, AXO, CHL, FIS, FOX, STR, TET, XNL	AMP, FFN, SDM, TET, XNL	+
RM012	17 September 2001	3/157	Rope (R1)	Uganda	AMP, AUG2, AXO, CHL, FIS, FOX, STR, TET, XNL	AMP, FFN, SDM, TET, XNL	-
RM013	17 September 2001	3/157	Rope (R1)	Uganda	AMP, AUG2, AXO, CHL, FIS, FOX, STR, TET, XNL	AMP, FFN, SDM, TET, XNL	+
RM014	17 September 2001	3/157	Rope (R4)	Typhimurium	AMP, AUG2, AXO, CHL, FIS, STR, TET, XNL	AMP, FFN, SDM, TET, XNL	+
RM015	17 September 2001	3/157	Rope (R4)	Typhimurium	AMP, AUG2, AXO, CHL, FIS, FOX, STR, TET, XNL	AMP, FFN, SDM, TET, XNL	+
RM016	17 September 2001	3/157	Water (W2)	Uganda	AMP, AUG2, AXO, CHL, FIS, FOX, STR, TET, XNL	AMP, FFN, SDM, TET, XNL	+
RM017	17 September 2001	3/157	Water (W2)	Uganda	AMP, AUG2, AXO, CHL, CIP, FIS, GEN, STR, TET, XNL	AMP, FFN, SDM, TET, XNL	+
RM018	17 September 2001	3/157	Water (W2)	Uganda	AMP, AUG2, AXO, CHL, FIS, FOX, STR, TET, XNL	AMP, FFN, SDM, TET, XNL	+
RM019	23 October 2001	2/822b	Rope (R1)	Give	AMP, AUG2, AXO, CHL, FIS, STR, TET, XNL	Pan-susceptible	+

^1^ Feedyard and lot numbers correspond to feedyard and respective lot within the feedyard from which the sample originated [25]. ^2^ Sample: W = water from tanks in pens; F = feces from pen floor; R = manila ropes placed above water tanks and feed bunks used as sampling devices. The number following W, F, or R was the sample identification number from the respective pen [25]. ^3^ Phenotypic AMR profile in NARMS 2006 assay. NARMS: U.S. National Antimicrobial Resistance Monitoring System; (2006) refers to year in which isolate was tested using the NARMS protocol. Antimicrobials tested and abbreviations: AMI = Amikacin; AMP = Ampicillin; AUG2 = Amoxicillin/clavulanic acid; AXO = Ceftriaxone; CHL = Chloramphenicol; CIP = Ciprofloxacin; FIS = Sulfasoxazole; FOX = Cefoxitin; GEN = Gentamicin; KAN = Kanamycin; NAL = Nalidixic acid; STR = Streptomycin; SXT = Trimethoprim/sulfamethoxazole; TET = Tetracycline; XNL = Ceftiofur. Multidrug resistant (MDR) isolates (resistant to ≥3 antibiotic classes) based on NARMS 2006 assay results are shaded in gray. ^4^ Phenotypic AMR profile in Sensititre Bovine BOPO6/7F (BOPO6F or BOPO7F, Thermo Fisher Scientific Waltham, MA, USA) assay (https://www.thermofisher.com, accessed on 3 November 2020); 2020 refers to year in which isolate was tested; antimicrobial abbreviations: AMP = Ampicillin; CLI = Clindamycin; DANO = Danofloxacin; ENRO = Enrofloxacin; FFN = Florfenicol; GAM = Gamithromycin; GEN = Gentamicin; NEO = Neomycin; PEN = Penicillin; SDM = Sulfadimethoxine; SPE = Spectinomycin; TET = Tetracycline; TIA = Tiamulin; TIL = Tilmicosin; TIP = Tildipirosin; TUL = Tulathromycin; TYLT = Tylosin tartrate; XNL = Ceftiofur. MDR isolates based on BOPO7F (2020) results are shaded in yellow. ^5^ ND: Not done. ^6^ WGS: Whole-genome sequencing was performed.

**Table 2:** MDR *Salmonella enterica* isolates from cattle systems accessions to the NVDC during the period of 2011–2020.

Isolate	Date	Signalment	History/Pathology	Sample ^1^	Serotype ^2^	AMR Phenotype ^3^	WGS ^4^
RM093	12 September 2019	Cow	Diarrhea, hepatitis	Small and large intestines	Anatum	DANO, ENRO, FFN, SDM, OXY, XNL	-
RM043	20 May 2015	Calf	Bloody diarrhea, pneumonia, septicemia	Pool of small intestine and lung	Dublin	CTET, FFN, OXY, SDM, XNL	+
RM052	4 April 2017	No information	Pneumonia	Lung	Dublin	CTET, FFN, OXY, SDM	+
RM079	20 July 2018	Dairy calf	Pneumonia	Lung	Dublin	CTET, FFN, OXY, SDM, XNL	+
RM092	26 July 2019	Calf (9–10 week-old)	Pneumonia, septicemia	Lung	Dublin	ENRO, FFN, OXY, SDM, XNL	+
RM094	17 September 2019	Dairy calf (1–4 week-old)	Septicemia, pneumonia	Liver, lung, small intestine	Dublin	DANO, ENRO, FFN, OXY, XNL	+
RM099	7 November 2019	Calf	Diarrhea, pneumonia	Pooled lung, small and large intestines	Dublin	DANO, ENRO, FFN, OXY, XNL	+
RM100	15 January 2020	No information	No information	Bacterial isolate	Dublin	DANO, ENRO, FFN, SDM, TET, XNL	+
RM104	3 April 2020	Calf (1-week-old)	Septicemia, pneumonia	Feces	Dublin	FFN, OXY, SDM, XNL	+
RM105	7 April 2020	Calf	Septicemia, pneumonia	Lung and small intestine	Dublin	FFN, OXY, SDM, XNL	+
RM111	11 June 2020	No information	Diarrhea, pneumonia	Feces	Dublin	FFN, OXY, SDM, XNL	+
RM101	12 February 2020	Calf (3–7-day-old)	Diarrhea, enteritis	Large intestine	Heidelberg	DANO, ENRO, OXY, SDM, XNL	+
RM055	11 May 2017	No information	No information	Feces	Muenster	DANO, ENRO, FFN, SDM	+
RM074	16 May 2018	No information	Diarrhea	Feces	Muenster	DANO, ENRO, FFN, SDM	+
RM037	10 April 2015	No information	No information	Feces	Newport	CTET, FFN, OXY, SDM, XNL	+
RM038	14 April 2015	Feedlot cattle	Bloody diarrhea	Feces	Newport	CTET, FFN, OXY, SDM, XNL	+
RM041	28 April 2015	Feedlot cattle	Bloody diarrhea	Feces	Newport	CTET, FFN, OXY, SDM, XNL	+
RM049	15 October 2015	Weaned calf	Bloody diarrhea	Feces	Newport	CTET, FFN, OXY, SDM, XNL	+
RM089	13 June 2019	Calf	No information	Feces	Newport	FFN, OXY, SDM, XNL	+
RM106	14 April 2020	Neonatal calf	Diarrhea, colitis	Large intestine	Saintpaul	FFN, OXY, SDM	+
RM086	5 April 2019	Calf	Diarrhea	Feces	Species	DANO, ENRO, FFN, OXY, SDM	-
RM095	9 October 2019	Bull (3-year-old)	Abomasitis, duodenitis, peritonitis (septicemia)	Liver, gallbladder, lymph node	Typhimurium	FFN, OXY, SDM, XNL	+

^1^ Sample from which *Salmonella* was isolated. ^2^ Isolates are sorted in the table by serotype. The isolate listed as *Salmonella* species was non-typeable. ^3^ AMR Phenotype: Antimicrobial Resistance Phenotype, based on results of Sensititre BOPO6F or BOPO7F testing. Antimicrobial abbreviations: AMP = Ampicillin; CTET = Chlortetracycline; DANO = Danofloxacin; ENRO = Enrofloxacin; FFN = Florfenicol; GEN = Gentamicin; NEO = Neomycin; OXY = Oxytetracycline; PEN = Penicillin; SDM = Sulfadimethoxine; SPE = Spectinomycin; TET = Tetracycline; TYLT = Tylosin tartrate; XNL = Ceftiofur. Resistance to macrolides (clindamycin, gamithromycin, tiamulin, tildipirosin, tilmicosin, tulathromycin, and tylosin tartrate) was considered intrinsic, and, therefore, not listed in the table. Multidrug resistance (MDR) is based on resistance to ≥3 antibiotic classes. ^4^ WGS: Whole-genome sequencing.

**Table 3:** Number of AMR genes associated with IS*Vsa3* and their locations in *Salmonella enterica* isolates as detected by WGS.

Number of AMR Genes ^1^	Isolates Positive ^2^	Plasmid ^3^	IncC Plasmid ^4^	*bla*_CMY-2_ Positive Plasmid		
				Isolates Positive ^5^	Plasmid ^6^	IncC Plasmid ^7^
1	7/41 (17.0%)	6/7 (85.7%)	0/6 (0.0%)	0/6 (0.0%)	NA	NA
2	0/41 (0.0%)	NA	NA	NA	NA	NA
3	0/41 (0.0%)	NA	NA	NA	NA	NA
4	3/41 (7.3%)	3/3 (100.0%)	1/3 (33.3%)	1/3 (33.3%)	1/3 (33.3%)	1/3 (33.3%)
5	26/41 (63.4%)	24/26 (90.0%)	19/24 (79.2%)	22/26 (84.6%)	21/22 (95.4%)	19/21 (90.5%)

^1^ Number of AMR genes detected (1 or more of the following) in association with IS*Vsa3*: *floR*, *tet(A)*, *aph(6)-Id*, *aph(3)-Ib*, and *sul2*. ^2^ Number of isolates positive for the respective number of AMR genes/number of isolates tested (percentage). NA = Not applicable. A total of 5 of 41 isolates tested did not have AMR genes in association with IS*Vsa3*. ^3^ Number of isolates positive for the respective number of AMR genes with a location on a plasmid/number of isolates positive for these genes (percentage). NA = Not applicable. ^4^ Number of isolates positive for the respective number of AMR genes with a location on an IncC plasmid/number of isolates positive for these genes (percentage) on a plasmid. NA = Not applicable. ^5^ Number of isolates positive for the respective AMR genes with concurrent presence of a *bla*_CMY-2_ positive plasmid/number of isolates positive for these genes (percentage). NA = Not applicable. ^6^ Number of isolates positive for the respective number of AMR genes with a location on a *bla*_CMY-2_ positive plasmid/number of isolates positive for these genes (percentage) and containing a *bla*_CMY-2_ positive plasmid. NA = Not applicable. ^7^ Number of isolates positive for the respective number of AMR genes with a location on a *bla*_CMY-2_ positive IncC plasmid/number of isolates positive for these genes (percentage) on a *bla*_CMY-2_ positive plasmid. NA = Not applicable.

**Table 4:** Frequency of IS*Vsa3*, associated AMR genes, and their genomic locations as detected by WGS.

Gene	Isolates Positive ^1^	Plasmid ^2^	IncC Plasmid ^3^
IS*Vsa3*	36/41 (87.8%)	33/36 (91.7%)	20/33 (60.6%)
*floR*	30/36 (73.2%)	27/30 (90.0%)	20/27 (74.1%)
*tet(A)*	27/36 (65.9%)	25/27 (92.6%)	20/25 (80.0%)
*aph(3″)-Ib*	29/36 (70.7%)	27/29 (93.1%)	20/27 (74.1%)
*aph(6)-Id*	28/36 (68.3%) ^4^	26/28 (92.9%) ^4^	19/26 (73.1%) ^4^
*sul2*	35/36 (87.8%)	33/36 (91.7%)	19/33 (57.6%)
*bla*_CMY-2_	25/36 (61.0%) ^5^	23/25 (92.0%) ^5^	20/23 (87.0%) ^5^

^1^ In column, data in the first cell refer to number of isolates positive for IS*Vsa3*/number of isolates tested (percentage). In the remainder of the column, the data refer to the number of IS*Vsa3*-positive isolates positive for gene/number of IS*Vsa3*-positive isolates tested (percentage). ^2^ For the entire column, the data refer to number of IS*Vsa3*-positive isolates in which the gene was located on a plasmid/number of IS*Vsa3*-positive isolates that were positive for the gene (percentage). ^3^ For the entire column, data refer to number of IS*Vsa3*-positive isolates in which the gene was located on an IncC plasmid/number of IS*Vsa3*-positive isolates positive for the gene on a plasmid (percentage). ^4^ Positive WGS results for either the *aph(6)-Id* or *aph(6)-I* family genes as shown in Tables S6 and S7 were counted as positive for *aph(6)-Id*. ^5^ Isolate RM043 has 2 copies of *bla*_CMY-2_, but only 1 copy was counted in the total, as shown in Table S6.

**Table 5:** A 2 × 2 contingency table showing association between MDR phenotype and IS*Vsa3* genotype with OR calculation.

MDR Phenotype ^1^				
**IS*Vsa3* Genotype ^2^**		**Positive**	**Negative**	**Total**
	**Positive**	31	6	37
	**Negative**	2	72	74
	**Total**	33	78	111
**OR ^3^**	OR = [(31 × 72)/(6 × 2)]/12 = 2232/12 = 186.00 (CI = 35.55–973.15; z statistic = 6.190; *p* < 0.0001)			

^1^ MDR isolates (MDR phenotype positive) were resistant to ≥3 antibiotic classes, whereas non-MDR (MDR phenotype negative) isolates were resistant to 0–2 antibiotic classes. ^2^ IS*Vsa3* genotype included the combined results of WGS and PCR. ^3^ OR = Odds ratio, testing association between MDR phenotype and IS*Vsa3* in 111 *S. enterica* isolates, determined using MedCalc^®^ statistical software, Version 20.218; CI = 95% confidence interval; *p* < 0.05 is statistically significant.

## Data Availability

All WGS data on the 41 isolates is available under NCBI BioProject PRJNA929056. All other data are available within this article and the supplementary materials.

## Supplementary Materials

The following supporting information can be downloaded at: https://www.mdpi.com/article/10.3390/microorganisms11030631/s1, Table S1. Serotypes of *Salmonella* isolates from cattle in accessions submitted to the Nebraska Veterinary Diagnostic Center from 2011 to 2020; Table S2. Frequency of association of *Salmonella* isolation with bovine source and disease manifestation in Nebraska Veterinary Diagnostic Center accessions; Table S3. List of *Salmonella* isolates from Nebraska Veterinary Diagnostic Center arranged in chronological order of isolation with accompanying information concerning signalment, clinical history, sample type, serotype, and antimicrobial resistance phenotype; Table S4. Primers, probes, and products of multiplex qPCR assays; Table S5. Primer pairs and products used in endpoint PCR assays; Table S6. Antibiograms, mqPCR, endpoint PCR, and whole-genome sequencing results of *Salmonella enterica* isolates (*n* = 111)**;** Table S7. Combined endpoint PCR and WGS results for IS*Vsa3*, *bla*_CMY-2_, *floR*, *tet(A)*, *sul2*, *aph(3″)-Ib*, and *aph(6)-Id* on *Salmonella enterica* isolates (*n* = 111); Figure S1. Alignment of the genetic segment of the 36 sequenced isolates carrying IS*Vsa3* and associated AMR genes.
