# Supplementary material for: Association of ISVsa3 with Multidrug Resistance in Salmonella enterica Isolates from Cattle (Bos taurus)
Source: Microorganisms. 2023 Mar 1;11(3):631. doi: 10.3390/microorganisms11030631 (PMC10051122; doi:10.3390/microorganisms11030631)
Supplement: Supplementary file 1 [file microorganisms-11-00631-s001.zip › Table S1.pdf]

Table S1. Serotypes of *Salmonella* isolates from cattle in accessions submitted to the Nebraska Veterinary Diagnostic Center from 2011-2020.

Serotype	Number of Isolates (%)
Typhimurium/3 var. 5-	18 (19.6%)
Newport	13 (14.1%)
Dublin	10 (10.9%)
Montevideo	8 (8.7%)
Muenster	7 (7.6%)
Anatum	4 (4.3%)
Muenchen	4 (4.3%)
Oranienburg	3 (3.3%)
4,[5],12:i:-	2 (2.2%)
Agona	2 (2.2%)
Idikan	2 (2.2%)
Orion/Orion var. 15+	2 (2.2%)
Schwarzengrund	2 (2.2%)
Enteritidis	1 (1.1%)
Heidelberg	1 (1.1%)
Infantis	1 (1.1%)
Kiambu	1 (1.1%)
Litchfield	1 (1.1%)
Liverpool	1 (1.1%)
Mbandaka	1 (1.1%)
Minnesota	1 (1.1%)
Rough O:K:1,5	1 (1.1%)
Saintpaul	1 (1.1%)
Senftenberg	1 (1.1%)
Soerenga	1 (1.1%)
Thompson	1 (1.1%)
Worthington	1 (1.1%)
