# Supplementary material for: Association of ISVsa3 with Multidrug Resistance in Salmonella enterica Isolates from Cattle (Bos taurus)
Source: Microorganisms. 2023 Mar 1;11(3):631. doi: 10.3390/microorganisms11030631 (PMC10051122; doi:10.3390/microorganisms11030631)
Supplement: Supplementary file 1 [file microorganisms-11-00631-s001.zip › Table S2.pdf]

Table S2. Frequency of association of *Salmonella* isolation with bovine source and disease manifestation in Nebraska Veterinary Diagnostic Center accessions.

Factor	Number of Isolates[1]	Percentage of Isolates[1]
Signalment available	84	91.3
Clinical history and/or pathology data available	78	84.8
Source of isolate		
Fetus	8	8.7
Cow/heifer	27	29.3
Bull	3	3.3
Feedlot/weaned	8	8.7
Young unweaned/neonatal calf	35	38.0
Environmental	1	1.1
No/insufficient information	10	10.9
Disease[2]	75	81.5
Abortion	10	10.9
Diarrhea/enteritis/colitis	49	53.3
Pneumonia	19	20.7
Septicemia	15	16.3

[1]From a total of 92 isolates.

[2]Disease manifestations in cattle from which *Salmonella* was isolated. Some animals had >1 disease manifestation (e.g., both pneumonia and septicemia), and both were counted. Aborted fetuses with pneumonia were counted only as abortion. In 2 accessions, the significance of *Salmonella* isolation in a case of pneumonia could not be determined since lungs were coinfected with other primary respiratory pathogens. In 2 accessions, the significance of *Salmonella* isolation as an enteric pathogen could not be determined since the animal was coinfected with *Mycobacterium paratuberculosis*.
