# Supplementary material for: Association of ISVsa3 with Multidrug Resistance in Salmonella enterica Isolates from Cattle (Bos taurus)
Source: Microorganisms. 2023 Mar 1;11(3):631. doi: 10.3390/microorganisms11030631 (PMC10051122; doi:10.3390/microorganisms11030631)
Supplement: Supplementary file 1 [file microorganisms-11-00631-s001.zip › Table S5.pdf]

Table S5. Primer pairs and products used in endpoint PCR assays.

Primer	Sequence	Amplicon (bp)	Reference
Single-plex PCR			
APH(3'')-Ib-3F	GTCAGAGGCGGAGAATCTGG	729	This study
APH(3'')-Ib-3R	TCGCAGATAGAAGGCAAGGC		
APH(6)-Id-3F	ACTCCTGCAATCGTCAAGGG	721	This study
APH(6)-Id-3R	TCTGTCGCACCTGCTTGATC		
CMY-F	CCGGACACCTTTTTGCTTTT	1205	[30]
CMY-R	TATCCTGGGCCTCATCGTCAGTTA		
CMY-2F	TTCTCCGGGACAACTTGACG	188	[31]
CMY-2R	GCATCTCCCAGCCTAATCCC		
IS*Vsa3*-3F	TGGAGCATGGCTTTCTACGG	951	This study
IS*Vsa3*-3R	GGGTTAGGTTGACTCTGGGC		
tetA-3F	TGTCGACTACGCCATCATGG	895	This study
tetA-3R	CAGGCAGAGCAAGTAGAGGG		
Duplex PCR			
ICEFloR-F	CGATCTGTCTTGCCGATCTT	84	[27]
ICEFloR-R	GACGAAGAAGGTGCCCATAC		
ICESul2-F	CGTTATGCATTCGGTGCAAG	135	[27]
ICE Sul2-R	AGGCGGTTGCGTTTGATA		
Three-plex PCR			
ICEFloR-F	CGATCTGTCTTGCCGATCTT	84	[27]
ICEFloR-R	GACGAAGAAGGTGCCCATAC		
ICESul2-F	CGTTATGCATTCGGTGCAAG	135	[27]
ICE Sul2-R	AGGCGGTTGCGTTTGATA		
IS*Vsa3*-3F	TGGAGCATGGCTTTCTACGG	951	This study
IS*Vsa3*-3R	GGGTTAGGTTGACTCTGGGC		
